# Prevalence of different comorbidities in chronic obstructive pulmonary disease among Shahrekord PERSIAN cohort study in southwest Iran

**DOI:** 10.1038/s41598-020-79707-y

**Published:** 2021-01-15

**Authors:** Fatemeh Zeynab Kiani, Ali Ahmadi

**Affiliations:** 1 Modeling in Health Research Center, Shahrekord University of Medical Sciences, Shahrekord, Iran; 2 Department of Epidemiology and Biostatistics, School of Health and Modeling in Health Research Center, Shahrekord University of Medical Sciences, Shahrekord, Iran

**Keywords:** Chronic obstructive pulmonary disease, Outcomes research

## Abstract

Comorbidities are common in chronic obstructive pulmonary disease (COPD) patients. This study was conducted to determine the prevalence of common comorbidities in patients with COPD compared with people without COPD. This cross-sectional, population-based study was performed on 6961 adults aged 35–70 years enrolled in the Shahrekord PERSIAN cohort study. Data (demographic and clinical characteristics, comorbidities, anthropometric and blood pressure measurements, laboratory, and spirometry tests) collection was performed according to the cohort protocol from 2015 to 2019. In the present study, 215 (3.1%) patients were diagnosed with COPD and 1753 (25.18%) ones with restrictive lung patterns. The mean age of COPD patients was 52.5 ± 9.76 years. 55.8% of patients were male, 17.7% were current smokers and 12.1% had a history of smoking or were former smokers. 5.6% of patients had no comorbidity and 94.5% had at least one comorbidity. The most common comorbidities in COPD patients were dyslipidemia (70.2%), hypertension (30.2%), metabolic syndrome (22.8%), and diabetes (16.7%). The most common comorbidities in individuals with a restrictive spirometry pattern were dyslipidemia (68.9%), metabolic syndrome (27.2%), hypertension (26.1%), depression (17.6%), and fatty liver (15.5%). The logistic regression analysis with 95% confidence interval (95%CI) of odds ratio (OR) showed that comorbidities of chronic lung diseases (OR = 2.12, 95% CI 1.30–3.44), diabetes (OR = 1.54, 95%CI 1.03–2.29), cardiovascular disease (OR = 1.52, 95%CI 1.17–2.43), and hypertension (OR = 1.4, 95%CI 1.02–1.99) were more likely to occur in COPD patients than in healthy individuals. Knowing these prevalence rates and related information provides new insights on comorbidities to reduce disease burden and develop preventive interventions and to regulate health care resources to meet the needs of patients in primary health care.

## Introduction

Chronic obstructive pulmonary disease (COPD) is defined as a group of chronic pulmonary inflammatory disorders characterized by persistent airflow restriction. COPD is one of the leading causes of death worldwide and is projected to be the third leading cause of death in 2020. Comorbidities are common in patients with COPD, substantially affect the patients' prognosis, quality of life, and survival, and are more common among disadvantaged social groups^1–3^.

Like other chronic diseases, COPD is associated with comorbidities, whose number and severity increase with increasing age^4,5^. It is estimated that at least 80% of COPD patients have at least one comorbidity^6–8^. A number of non-communicable diseases (NCDs) often occur as clusters of comorbidities in patients with COPD^9^. Studies have shown that comorbidities mainly include heart failure, cardiovascular disease, metabolic syndrome, diabetes, anxiety/depression, and osteoporosis^5,10–12^.

Comorbidities are very important in COPD patients for several reasons. There are shared pathophysiological mechanisms in COPD and other chronic diseases. For example, some comorbidities may have a substantial impact on health and healthcare services use, leading to increased severity and hospitalization of COPD patients and some others such as heart disease can lead to death earlier than respiratory causes^13^.

The association between COPD and these comorbidities may be explained by common risk factors, such as smoking, aging, and physical inactivity^14,15^.

Determining the actual prevalence of comorbidities in COPD patients and its association with COPD severity may be difficult due to no diagnosis or underdiagnosis of COPD, common risk factors for both COPD and comorbidities, lack of diagnosis of comorbidities, and characteristics of comorbidities that may overlap with the characteristics used to define the severity of COPD^16,17^. The prevalence of comorbidities in patients with COPD is thought to be high. No studies have yet been performed in Iran to determine the prevalence of comorbidities in patients with COPD compared with people without COPD in a wide age range. Therefore, this study is the first study that was conducted comprehensively in southwestern Iran to determine the prevalence of common comorbidities in patients with COPD compared with those without COPD.

## Methods

The current study used the data of the Shahrekord cohort study (SCS) with a population-based cross-sectional design in the baseline recruitment phase. SCS^18^ is a part of the PERSIAN (Prospective Epidemiological Research Studies in IrAN) cohort^19^ with a sample size of 10,075 adults in Shahrekord, Iran. This cohort study is aimed to investigate the incidence and prevalence of non-communicable diseases and improve lifestyle in Chaharmahal and Bakhtiari province, southwestern Iran. The protocol, sampling, laboratory measurements, and physical examinations of the SCS have already been published^18,19^. Data (demographic and clinical characteristics, comorbidities, anthropometric and blood pressure measurements, laboratory, and spirometry tests) collection was performed according to the cohort protocol from 2015 to 2019.

### Instruments

We used demographic and clinical characteristics checklist including age, sex, education level, marital status, body mass index (BMI), smoking, respiratory symptoms (such as shortness of breath, cough, sputum, and wheezing)^18,19^.

The status of smoking was investigated by two questions: *Have you smoked at least 100 cigarettes in your entire life*? and *Do you smoke now*? Participants could answer the first question with *Yes* or *No* and the second question with *Yes, every day*; *Yes, sometimes*; and *No*. Those who answered both questions were selected as non-smokers. Those who answered both questions were selected as non-smokers. Those who answered *Yes* to the first question but *No* to the second answer were considered as people with a history of smoking or former smokers (an individual who has smoked at least 100 cigarettes in his/her lifetime but who had quitted smoking before interview).

Studied comorbidities were hypertension, diabetes, metabolic syndrome, dyslipidemia, cardiovascular disease, chronic lung disease other than COPD, depression, osteoporosis, and fatty liver. Participants were asked to answer whether any of these comorbidities were diagnosed by a doctor or if they were taking medication or being treated.

Hypertension was defined as systolic blood pressure above 140 mmHg or diastolic blood pressure above 90, or having previously been diagnosed by a physician or taking antihypertensive drugs^20^.

Diabetes was defined as fasting blood levels above 126 mmHg or having previously been diagnosed by a physician or taking antihyperglycemic drugs^21^.

The metabolic syndrome was defined according to *the ATP Adult treatment panel* as follows: People who had three or more of the following criteria were considered as suffering from metabolic syndrome: Abdominal obesity (waist circumference for men ≥ 102 cm, women ≥ 88 cm); TG ≥ 150 mg/dL; HDL-c < 40 mg/dL for men and HDL-c < 50 mg/dL for women or drug treatment for low HDL-c; SBP ≥ 130 mmHg or DBP ≥ 85 mmHg or receiving antihypertensive treatment with a history of hypertension and fasting blood sugar levels ≥ 100 mg/dL or receiving drug therapy to increase glucose levels^22^.

According to ATP III, dyslipidemia was defined as having one or more of the following disorders: Total cholesterol ≥ 200, TG ≥ 150 mg/dL, LDL ≥ 130 mg/dL, HDL-c < 40 mg/dL for men and HDL-c < 50 mg/dL for women^11^. Cardiovascular diseases were investigated by asking if *you have a history of any myocardial infarction, heart failure, angina, or transient ischemic attack or stroke*.

The presence of chronic lung disease was investigated by asking *If any of the following conditions have been diagnosed by a doctor or healthcare worker: tuberculosis, asthma, emphysema, chronic bronchitis*. The presence of cough, sputum, shortness of breath and wheezing was investigated by questions such as *Have you had a cough for three consecutive months or more in the last year*?", *Have you had sputum for three consecutive months or more during the past year*? *Have you had shortness of breath for the past 3 months when rushing on the surface or walking on uneven surfaces*? and *Have you ever felt suffocated or whistling in your chest*?

Depression was investigated by the question *How often do you experience problems such as feelings of failure, depression, or frustration over the past 2 weeks*? Participants could answer this question by (1) *Never ever*, (2) *More than half a day*; (3) *A few days,* and (4) *Almost every day*.

### COPD diagnosis

For all participants, pre-bronchodilator spirometry was performed using a portable spirometer (New Spirolab, MIR, Italy, 2015) between 8 a.m. and 2 p.m.; all tests were conducted according to the ATS/ERS guidelines (the American Thoracic Society/European Respiratory Society) in a quiet room in a sitting position on a comfortable chair^23^.

The spirometer was calibrated using a syringe of 3 l by trained technicians daily before the study began. The spirometer was considered calibrated when injecting 3 l of air into the device; the error rate was not more than 3% or 90 cc (registration number should be between 2.91–3.09 L). Technicians performing spirometry were trained through a special training course in a pulmonary function laboratory in the hospital.

All participants were informed about all stages in the investigation and the pulmonary function test. All steps of the spirometry maneuver were performed practically by the technician so that the participant could see how to do proper inhaling and exhaling. Spirometry was performed three times for each subject according to the same conventional manner.

By comparing the curves of these three pulmonary function tests, the maximal values for forced vital capacity (FVC), forced expiratory volume in 1 s (FEV1), Maximum Ventilatory Volume (MMV), and maximum peak expiratory flow (PEF) in 75%, 50% and 25% of FVC (PEF25-75) were obtained from the three curves.

A participant must have at least two acceptable curves with repeatable FVC and FEV1 values in 250 ml. Spirometry data were interpreted according to the ATS/ERS recommendations by two respiratory medicine specialists. The values of pulmonary function test parameters were presented as the percent of predicted values for the respective age, height, and weight. Contraindications to spirometry included uncontrolled hypertension above 140/100, myocardial infarction, pulmonary embolism, diagnosed aneurysms, recent surgery on the eyes, ears, brain, abdomen and chest, liver, heart or kidney failure, cancer and endocrine disorders).

COPD was defined as a fixed ratio FEV1/FVC < 70% according to the Global Initiative for Obstructive Lung Disease (GOLD) guidelines. COPD was defined as a fixed ratio FEV1/FVC > 70 according to the GOLD guidelines and restrictive spirometry was defined as FEV1/FVC > 0.70 but FEV1 or FVC < 80% (%)^3^.

The whole procedure of data collection was monitored by a quality control team, including clinicians, a laboratory specialist, two statisticians, and an epidemiologist, under the supervision of the principal investigators.

### Statistical analysis

All continuous quantitative variables were expressed as mean ± standard deviation and categorical variables as frequency and percentage. The chi-square test was used to compare the frequency of comorbidities between people with and without COPD. Logistic regression analysis adjusted for age, sex, education level, body mass index, and smoking status was carried out to determine the likelihood of comorbidities [(odds ratios (OR) with 95% confidence interval (CI)] in people with and without COPD. *P* < 0.05 was considered significance level. Data analysis was performed using stata software (Stata Corp. 2015. Stata Statistical Software: Release 14. College Station, TX: Stata Corp LP.). All measures were reported by all subjects, COPD patients, individuals with restrictive spirometry, and healthy individuals (non- COPD), as well as by gender among individuals with and without COPD.

### Ethics approval and consent to participate

This study was conducted with observance of the Declaration of Helsinki and the National Ethical Guidelines in Biomedical Research in Iran. As well, the study protocol was approved by the Ethics Committee of the SKUMS (IR.SKUMS.REC 1394.286 and IR. SKUMS.1396.110) at regional and national scales. All participants provided signed and fingerprinted informed written consent according to the Guidelines enforced by the Ethics Committee of the SKUMS. The participants can withdraw from the study whenever they wish. Data are stored in a codified confidential database.

## Results

In the current study, of the 6961 participants, 3299 (47.4%) were male**,** 3662 (52.6%) were female and 6572 (94.4%) were married. The mean ± standard deviation (SD) of age for COPD patients, individuals with restrictive spirometry, and healthy individuals were 52.5 ± 9.7, 50.1 ± 9.4 and 49.05 ± 9.1 respectively. The prevalence of smoking in all men and women was 29.7% (n: 980) and 0.4% (n: 14), respectively. The prevalence of current smoking in the individuals with COPD was 17.7% (30.8% in men and 1.05% in women) and the prevalence of formerly smoking was 12.1%, respectively.

5.6% of patients had no comorbidities and 94.5% had at least one comorbidity. In the present study, 215 (3.1%) patients were diagnosed with COPD and 1753 (25.18%) patients with restrictive lung patterns (Table 1).

**Table 1:** Baseline characteristics of the Shahrekord cohort study; sub-cohort of COPD.

Characteristic	Total subjects N = 6961	COPD (n = 215)	FEV1/FVC > 0.70 but FEV1 or FVC < 80% (%), (n = 1753)	Non-COPD (n = 4993)	P-value
Age (year), mean ± SD	49.44 ± 9.29	52.50 ± 9.76	50.14 ± 9.48	49.05 ± 9.17	< 0.0001
FEV1 (% pred), mean ± SD	100.92 ± 14.67	78.75 ± 18.36	80.79 ± 11.09	106.26 ± 15.19	< 0.0001
FVC (% pred), mean ± SD	88.71 ± 16.78	94.87 ± 24.12	70.75 ± 8.92	94.75 ± 13.72	< 0.0001
FEV1/FVC, mean ± SD	92.39 ± 7.48	68.49 ± 7.36	94.38 ± 6.15	92.71 ± 6.03	< 0.0001
BMI (kg/m^2^), mean ± SD	27.75 ± 4.58	28.12 ± 4.69	27.39 ± 5.03	27.86 ± 4.40	0.001
Education (N years), mean ± SD	8.87 ± 6	7.84 ± 6.15	7.17 ± 5.98	9.51 ± 5.91	< 0.0001
Gender, male, n (%)	3299 (47.4%)	120 (55.8%)	722 (41.2%)	2453 (49.2%)	< 0.0001
Gender, female, n (%)	3662 (52.6%)	95 (44.2%)	1031 (58.8%)	3534 (50.8%)	
Single, n (%)	108 (1.6%)	4 (1.9%)	34 (1.9%)	70 (1.4%)	< 0.0001
Married, n (%)	6572 (94.4%)	201 (93.5%)	1612 (92%)	4753 (95.3%)	
Widow and divorced, n (%)	281 (4%)	10 (4.7%)	107 (6.1%)	164 (3.3%)	
Ex-smoker, n (%)	546 (7.8%)	26 (12.1%)	106 (6.1%)	413 (8.3%)	< 0.0001
Current-smoker, n (%)	994 (14.3%)	38 (17.7%)	279 (15.9%)	676 (13.6%)	
Never-smoker, n (%)	5417 (77.9%)	151 (70.2%)	1367 (78%)	3895(78.2%)	
**Comorbidities and prevalence frequency (%)**					
0	938 (12.8%)	12 (5.6%)	142 (8.1%)	395 (7.9%)	0.743
1	1550 (21.1%)	52 (24.2%)	374 (21.3%)	1122 (22.5%)	
2	1763 (24%)	54 (25.1%)	438 (25%)	1271 (25.5%)	
≥ 3	3098 (42.2%)	97 (45.1%)	799 (45.6%)	2199 (44.1%)	

COPD: chronic obstructive pulmonary disease; n: frequency; FEV1: forced expiratory volume in 1 s; FVC: forced vital capacity; BMI: body mass index.

Table 2 presents the prevalence of common comorbidities among COPD patients, individuals with restrictive spirometry, and healthy individuals, as well as among individuals with and without COPD by gender.

**Table 2:** Prevalence of comorbidities in all subjects and comparison by COPD, FEV1/FVC and gender in Shahrekord cohort study-subcohort of COPD.

Comorbidities	Total subjects N = 6961	Categorized by COPD and FEV1/FVC				Gender							
						Males N = 3299				Female N = 3662			
Number and (%)	Total	COPD or FEV1/FVC < 0.70 N = 215	FEV1/FVC > 0.70 but FEV1 or FVC < 80% (%) N = 1753	Non-COPD FEV1/FVC > 0.70 N = 4993	P-value	COPD or FEV1/FVC < 0.70 N = 120	FEV1/FVC > 0.70 but FEV1 or FVC < 80% (%) N = 722	Non-COPD FEV1/FVC > 0.70 N = 2457	P-value	COPD or FEV1/FVC < 0.70 N = 95	FEV1/FVC > 0.70 but FEV1 or FVC < 80% (%) N = 1031	Non-COPD FEV1/FVC > 0.70 N = 2536	P-value
Hypertension	1666 (23.9%)	65 (30.2%)	457 (26.1%)	1141 (22.9%)	0.002	30 (25%)	158 (21.9%)	590 (24.1%)	0.460	35 (36.8%)	299 (29%)	551 (21.8%)	< 0.0001
Diabetes mellitus	861 (12.3%)	36 (16.7%)	251 (14.3%)	574 (11.4%)	0.001	16 (13.3%)	88 (12.1%)	279 (11.3%)	0.699	20 (21.1%)	163 (15.8%)	295 (11.6%)	< 0.0001
Syndrome metabolic	1822 (26.1%)	49 (22.8%)	478 (27.2%)	1294 (25.9%)	0.288	23 (19.2%)	143 (19.8%)	518 (21.1%)	0.679	26 (27.4%)	335 (32.5%)	776 (30.6%)	0.403
Dyslipidemia	4875 (70%)	151 (70.2%)	1208 (68.9%)	3513 (70.3%)	0.534	85 (70.8%)	447 (61.9%)	1646 (66.9%)	0.027	66 (69.4%)	761 (73.8%)	1867 (73.6%)	0.495
Cardiovascular disease	461 (6.6%)	24 (11.2%)	147 (8.4%)	290 (5.8%)	< 0.0001	18 (15%)	67 (9.3%)	183 (7.5%)	0.006	6 (6.3%)	80 (7.8%)	107 (4.2%)	< 0.0001
Depression	1195 (17.2%)	29 (13.5%)	308 (17.6%)	857 (17.2%)	0.655	9 (7.5%)	77 (10.7%)	219 (8.9%)	0.487	20 (21.1%)	231 (22.4%)	638 (25.2%)	0.394
Osteoporosis	638 (9.2%)	21 (9.8%)	186 (10.6%)	431 (8.6%)	0.047	4 (3.3%)	14 (1.9%)	35 (1.4%)	0.195	17 (17.9%)	172 (16.7%)	396 (15.6%)	0.649
Chronic lung disease (asthma, tuberculosis, emphysema and bronchitis)	331 (4.8%)	22 (10.2%)	116 (6.6%)	193 (3.9%)	< 0.0001	14 (11.7%)	49 (6.8%)	92 (3.8%)	< 0.0001	8 (8.4%)	67 (6.5%)	101 (4%)	0.002
Fatty liver	1167 (16.8%)	29 (13.5%)	271 (15.5%)	865 (17.4%)	0.082	12 (10%)	76 (10.5%)	348 (14.2%)	0.022	17 (17.9%)	195 (18.9%)	517 (20.4%)	0.527

COPD: chronic obstructive pulmonary disease; FEV: forced expiratory volume; FVC: forced vital capacity.

The most common comorbidities in patients with COPD were dyslipidemia (70.2%), hypertension (30.2%), metabolic syndrome (22.8%), and diabetes mellitus (16.7%).

The most common comorbidities in individuals with a *restrictive* spirometry pattern were dyslipidemia (68.9%), metabolic syndrome (27.2%), hypertension (26.1%), depression (17.6%), and fatty liver (15.5%).

The most common comorbidity in men with COPD was dyslipidemia (70.8%), followed by hypertension (25%), metabolic syndrome (23%), cardiovascular disease (15%), and diabetes mellitus (13.3%), and in women dyslipidemia (69.4%), followed by hypertension (36.8%), metabolic syndrome (27.4%), diabetes mellitus (21.1%), and depression (21.1%). The most common comorbidity in men with restrictive patterns was dyslipidemia (61.9%), followed by hypertension (21.9%), metabolic syndrome (19.8%), and diabetes (12.1%), and in women dyslipidemia (73.8%), followed by metabolic syndrome (32.5%), hypertension (29%), and depression (22.4%).

Among comorbidities, the prevalence of hypertension, diabetes, cardiovascular disease, and chronic lung disease was higher in COPD patients compared to healthy individuals and restrictive patients and in restrictive patients than healthy individuals (P < 0.05).

The prevalence of hypertension (30.2% vs. 26.1%), and diabetes (16.7% vs. 14.3%) in COPD patients was significantly higher than those with restrictive patterns. Moreover, the prevalence of hypertension (26.1% vs. 22.9%) and diabetes (14.3 vs. 11.4%) was significantly higher in people with restrictive pattern compared to healthy individuals.

The prevalence of hypertension and diabetes was significantly higher in women with obstructive pulmonary disease than in women with a restrictive and healthy pattern, but the difference was not significant in men (P > 0.05).

In the present study, there was no significant difference in the prevalence of dyslipidemia and metabolic syndrome among individuals with and without COPD, and individuals with restrictive patterns (P > 0.05).

The prevalence of cardiovascular disease (11.2% vs. 5.8%) and chronic lung disease (10.2% vs. 3.9%) was higher in people with COPD than in those without COPD. In addition, cardiovascular diseases (11.2% vs. 8.4%) and chronic lung diseases (10.2% vs. 6.6%) were significantly higher in COPD patients than in those with restrictive patterns. These differences were statistically significant in terms of gender.

The prevalence of depression was higher in individuals with restrictive spirometry patterns than in those with obstruction patterns (17.6% vs. 13.5%) but the difference was not statistically significant.

The prevalence of osteoporosis was significantly higher in patients with restrictive patterns than in those with obstructive patterns (10.6% vs. 9.8%), and in obstructive patients than in healthy individuals (9.8% vs. 8.6%). The difference, however, was not statistically significant in terms of gender (P > 0.05).

The prevalence of fatty liver was higher in restrictive pattern patients than in obstructive pattern patients, and higher in healthy individuals than in restrictive and obstructive patients, but the difference was not statistically significant. Regarding gender, the prevalence was significantly higher in healthy men than in patients with obstructive and restrictive patterns.

Table 3 presents the results of the logistic regression model with 95% confidence interval (95%CI) of odds ratio (OR) for different comorbidities after adjustment for age, sex, education level, body mass index, and smoking in people with COPD in comparison to those without COPD. The results showed that comorbidities of chronic respiratory and pulmonary diseases (OR = 2.12, 95% CI 1.30–3.44), diabetes (OR = 1.54, 95%CI 1.03–2.29), cardiovascular disease (OR = 1.52, 95%CI 1.17–2.43), and hypertension (OR = 1.4, 95%CI 1.02–1.99) were more likely to occur in COPD patients than in healthy individuals (p < 0.05).

**Table 3:** Odds ratio (OR) of co-morbid illness adjusted for age, gender, education level, body mass index and smoking status for COPD in Shahrekord cohort study.

Comorbidities^a^	Crud-OR (95% CI)	P-value	Adjusted OR (95% CI)	P-value
Hypertension	1.39 (1.03–1.87)	0.029	1.43 (1.02–1.99)	0.034
Diabetes mellitus	1.45 (1.01–2.09)	0.047	1.54 (1.03–2.29)	0.032
Syndrome metabolic	0.948 (0.475–1.36)	0.417	0.824 (0.406–1.19)	0.274
Dyslipidemia	1.01 (0.75–1.37)	0.916	1.14 (0.82–1.57)	0.422
Cardiovascular diseases	1.81 (1.27–2.80)	0.007	1.52 (1.17–2.43)	0.049
Depression	0.74 (0.50–1.10)	0.147	0.71 (0.47–1.1)	0.112
Osteoporosis	1.07 (0.68–1.69)	0.758	1.08 (0.67–1.73)	0.746
Chronic lung disease	2.37 (1.50–3.74)	< 0.0001	2.12 (1.30–3.44)	0.002
Fatty liver	0.768 (0.517–1.14)	0.192	0.686 (0.451–1.044)	0.079

OR: odds ratio; CI: confidence interval.

^a^For each comorbidity, reference group was considered to lack of comorbidity.

## Discussion

The present population-based cross-sectional study was performed to investigate the prevalence of common comorbidities in individuals with and without COPD. In patients with COPD, there is a substantial prevalence of comorbidities that can have a significant impact on the prognosis, severity, and even treatment of COPD^24^. However, this is a two-way association, and COPD is known to increase the prevalence and severity of these comorbidities. Besides that, it has been shown that comorbidities may be left undiagnosed in patients with COPD, which can challenge the treatment and management of COPD.

In the present study, 215 patients (3.1%) were diagnosed using the GOLD criterion (fixed ratio FEV1/FVC < 0.7). Accordingly, the prevalence of COPD in our study was lower than the prevalence reported in other countries and almost similar to that reported by Sharifi et al., who reported a 3.3% prevalence of COPD in the burden of obstructive lung disease in Iran^25^.

In another study conducted in five different geographical areas in Iran (north, south, west, and east of Iran), the overall prevalence of COPD was estimated at 4.9%. The prevalence of COPD was 2.8% in Mashhad, 3.7% in Mazandaran, 8.8% in Ahvaz, 4.1% in Tehran, and 13.9% in Kerman^26^. The prevalence of COPD was higher in our study than in the BOLD study including 14 countries, which reported the prevalence of COPD grade 2 to be over 1.8%^27^.

The difference in the prevalence of the disease between the present study and other studies may be due to the fact that the participants in this study are from the general population who may have better respiratory function than hospital patients. Another reason for the difference in the prevalence of COPD in Iran and other countries is the different pattern of smoking in Iran compared to other countries. Iran is a developing country with a much lower smoking rate than those in many developed countries^28^. Fourteen percent of Iranians use some kind of tobacco with a ratio of 6 to 1 man to woman^29^. It has also been reported that the prevalence of smoking is lower in Iranian women than those in other countries. In Iran, female smokers make up almost less than 5% of the population^30^. In one study in Iran, the overall prevalence of smoking was 12.7% (17.2% among men and 2.5% among women)^31^. In our study, 29.7% of men (980 people) and 0.4% of women (14 people) were smokers.

In addition, Chaharmahal and Bakhtiari province (our study setting) is geographically located about 2153 m above sea level and is known as the roof of Iran^18^. Various studies have reported the effect of altitude on the incidence and prevalence of COPD. Horner et al. in the PREPOCOL-PLATINO-BOLD-EPI-SCAN study argued that known risk factors were less frequent at high altitude and high altitude had no significant impact on COPD prevalence^32^. A PLATINO study in five Latin American countries found that at higher altitudes, the prevalence of COPD was lower^33^. A meta-analysis study of 80 articles published during 2003–2014 reported a high-altitude protective effect for COPD^34^. However, the effect of altitude on prevalence may be an example of an ecological fallacy and needs further investigation.

Another reason for the difference in the prevalence of COPD may be due to the very low level of environmental pollution and dust in Chaharmahal and Bakhtiari province, although no study has yet been conducted to study this argument.

In the present study, 5.6% of patients had no comorbidities and 94.5% had at least one comorbidity. The most common comorbidities in patients with COPD were dyslipidemia, hypertension, metabolic syndrome, and diabetes mellitus. Dyslipidemia, metabolic syndrome, hypertension, depression, and fatty liver were the most common comorbidities in individuals with a restrictive spirometry pattern. The most common comorbidity in men with COPD was dyslipidemia, followed by hypertension, metabolic syndrome, cardiovascular disease, and diabetes mellitus, and in women dyslipidemia, followed by hypertension, metabolic syndrome, diabetes mellitus, and depression. The most common comorbidity in people with restrictive patterns in men was dyslipidemia, followed by hypertension, metabolic syndrome and diabetes, and in women dyslipidemia, followed by metabolic syndrome, hypertension and depression**.** In the study of Sawalha et al., the most common comorbidities in men were cardiovascular disease and diabetes mellitus, while in women anxiety and depression. Hypertension is a disorder of lipid metabolism and obesity^35^. This finding also confirms the results of the García et al., showing that common comorbidities in 3183 people with COPD in Spain included diabetes, high blood pressure, lipid metabolism disorders, and obesity^36^. A study in Canada also showed that 95% of COPD patients had one or more comorbidities, with heart disease being the most common (75.1%) comorbidity^37^. Other studies have also shown that 80% of COPD patients have at least one comorbidity^6–8^. The results of a meta-analysis of 11 studies including a total of 47695183 patients with COPD, showed that common comorbidities including cardiovascular and cerebral diseases, endocrine and metabolic disorders, psychiatric and neurological disorders, gastrointestinal diseases, non-musculoskeletal disorders, and respiratory conditions other than COPD were significantly higher in patients with COPD than in those without COPD. In that study, including 262,014 COPD patients from three independent studies, 36% of patients had one type of comorbidity and 30% of patients had more than one type of comorbidity^12^. In the study of Negro, at least 76.6% of patients had one comorbidity, 68.8% had two comorbidities, and 47.9% had three or more comorbidities, and cardiovascular diseases were the most common disorders^38^. The review article of Corlateanu et al. indicated that the most common comorbidities among COPD patients were ischemic heart disease, hyperlipidemia, congestive heart failure, diabetes, metabolic syndrome, osteoporosis, and depression^10^ In a study conducted by Triest, atherosclerosis and dyslipidemia were the most common comorbidities in patients with COPD^39^. In the study of Mannino et al., which included 186,881 patients with COPD, the most common comorbidities were cardiovascular disease (34.8%), diabetes (22.8%), and asthma (14.7%). Most patients (52.8%) had one or two comorbidities^40^. The most common comorbidities in Serbia were hypertension (54.5%) and dyslipidemia (26.5%)^24^. The study of Matsumoto with data from the electronic medical records showed that the prevalence of airflow obstruction was 29.3% higher in patients with atherosclerosis than in patients with dyslipidemia (24.3%), diabetes mellitus (23.1%) or hypertension (27.1%)^41^. In a cohort study on 33,997 patients in North Carolina, five common diseases were hypertension (42%), shortness of breath (37%), heart disease (30%), diabetes (29%), and congestive heart failure (21%)^42^.

In the present study, the prevalence of hypertension and diabetes was higher in patients with COPD than in healthy individuals. Moreover, the prevalence of hypertension and diabetes was significantly higher in patients with chronic obstructive pulmonary disease than in those with restrictive patterns. Regarding gender, the prevalence of hypertension and diabetes was significantly higher in women with obstructive pulmonary disease than in women with restrictive and healthy patterns, but the difference was not significant for men. In the study of Tuleta et al., diabetes was significantly higher in patients with COPD than in Non-COPD patients. The prevalence of cardiovascular disease was about one-third of that in COPD patients. The distribution of hypertension and hypercholesterolemia was similar in the two groups^43^. In the present study, there was no significant difference in the prevalence of dyslipidemia and metabolic syndrome among individuals with and without COPD, and individuals with a restrictive pattern.

The prevalence of cardiovascular disease and chronic lung disease was significantly higher in COPD patients than in patients with restrictive patterns, and in patients with restrictive patterns than in healthy individuals. These differences remained significant in terms of gender. A possible reason for co-existence of COPD and cardiovascular comorbidities could be the involvement of specific inflammatory cells in the formation of atherosclerotic plaques and plaque rupture. The results of one study showed that COPD served as an independent predictor of atherosclerosis^44^. The results of a recent study showed that ischemic heart disease, congestive heart failure, and stroke were the most common CVDs in patients with COPD^45^. In the study of Eriksson et al., cardiovascular and hypertension comorbidities were more common in people with moderate to severe COPD than in people without COPD^46^. The study of Platino showed a significantly higher prevalence of asthma in respondents with COPD. Patients with COPD with respiratory failure and respiratory comorbidities showed a significantly increased risk of COPD outcomes compared to those without these comorbidities^42^.

The prevalence of depression was higher in people with restrictive spirometry patterns than in healthy individuals and people with obstruction patterns but the difference was not statistically significant, which is inconsistent with the study of Nagorni. They found that people with COPD were three times more likely to develop anxiety and depression than people without COPD^24^. The prevalence of osteoporosis was significantly higher in patients with restrictive patterns than in those with obstructive patterns, and in obstructive patients than in healthy individuals. The difference was not statistically significant in terms of gender (P > 0.05). Risk factors for osteoporosis in COPD can be due to long-term treatment with corticosteroids, overweight or old age. Romme et al. found that the prevalence of osteoporosis was high in patients with COPD, which is in agreement with our results^47^. Breyer et al. reported that the prevalence of osteoporosis in patients with COPD varied between 20 and 50% depending on BMI^48^. The prevalence of fatty liver was higher in restrictive patients than in obstructive patients, and higher in healthy individuals than in restrictive and obstructive patients but the difference was not statistically significant. Regarding gender, fatty liver was significantly higher in healthy men than in obstructive and restrictive patients.

The results of the logistic analysis showed that the probability of having chronic respiratory and pulmonary diseases was 2.12 times, diabetes mellitus was 1.54 times, cardiovascular comorbidities were 1.52 times and hypertension was 1.43 times higher (P < 0.05).

One of the strengths of this study was the use of data of the COPD subcohort population in Iran. One of the limitations of the present study was that some of the comorbidity variables were examined using a one-item questionnaire; furthermore, because the participants in our study were the general population and included individuals who may have a better respiratory function or mild COPD, our findings cannot be generalized to people with advanced COPD. Another limitation of the study was that due to its cross-sectional design, it was not possible to obtain causal conclusions.

## Conclusion

This study reported the prevalence of comorbidities. Knowing these prevalence rates and related information provides new insights on comorbidities to reduce disease burden and develop preventive interventions and to regulate health care resources to meet the needs of patients in primary health care. Managing and controlling comorbidities should be an important part of COPD prevention and management strategies that can improve the overall outcome. Therefore, COPD management programs should focus on the most common comorbidities.

## Data Availability

The study that is ongoing. The general information is available from: http://cohort.skums.ac.ir. All researchers across Iran and the world can have free access to the findings of this study, and necessary processes are available at the Cohort website to reproduce the research project, participate in collaborative research projects, and use the data. After requested, under conditions of collaboration and endowment, Access to the data is available for interest researchers from corresponding author in AA (aliahmadi2007@gmail.com).
